# Editorial: Real-world data and real-world evidence in lung cancer

**DOI:** 10.3389/fonc.2024.1436077

**Published:** 2024-06-07

**Authors:** Valerio Gristina, Chukwuka Eze

**Affiliations:** ^1^ Department of Precision Medicine in Medical, Surgical and Critical Care (Me.Pre.C.C.), University of Palermo, Palermo, Italy; ^2^ Department of Radiation Oncology, University Hospital, LMU Munich, Munich, Germany

**Keywords:** lung cancer, real-world data, real-world evidence, prognosis, survival

## 1 Introduction

Lung cancer remains the leading cause of cancer-related death worldwide in both sexes (1). This editorial compels attention to the critical need for real-world data (RWD) and real-world evidence (RWE) to augment lung cancer research. Traditional clinical trials, while essential, represent a highly controlled environment that may not fully translate to the complexities of everyday patient care (2, 3). RWD, in contrast, gathers information directly from real-world clinical needs and settings (4, 5). This unfiltered approach offers invaluable insights into how lung cancer treatments function in a broader patient population, encompassing factors like underlying health conditions and variations in treatment adherence. The editorial argues that RWE can illuminate crucial knowledge gaps, particularly for patient subgroups often excluded from traditional trials due to comorbidities or other factors (3). By incorporating this rich tapestry of real-life data, researchers and clinicians can develop more effective and comprehensive treatment strategies for the fight against one of the most lethal cancers.

## 2 Composition

The development of prediction models in the clinic to forecast long-term survival in early NSCLC is warranted, mostly considering the upcoming implementation of perioperative and adjuvant chemo-immunotherapy in such a highly heterogeneous disease. In a cohort study including 505 patients diagnosed with stage I-II NSCLC at a tertiary Spanish hospital, Torrente et al. developed a useful prognostic model based on easy-to-obtain clinical risk factors, identifying high- and low-risk patients to tailor adjuvant treatment while eventually adapting surveillance plans and avoiding unnecessary tests or visits. Namely, in patients with T2aN0 stage IB lung adenocarcinoma, adjuvant chemotherapy remains controversial. Lee et al. retrospectively observed an improved overall and cancer-specific survival only in tumors larger than 3 cm whereas no benefit was seen in smaller tumors even when harboring visceral pleural invasion. In a further analysis by Davey et al., the predictive value of peritumor density and dose variability on local relapse (LR) and regional failure (RF) following stereotactic ablative radiotherapy (SABR/SBRT) of NSCLC were assessed. An internal cohort of 199 patients and an external cohort of 76 patients for validation were analyzed. High peritumor density combined with high dose variability predicted LR but not RF. External validation confirmed the importance of this interaction. These findings suggest the potential use of this model to modify low-dose clinical target volume (CTV) margins for high-risk patients undergoing lung SABR.

Cai et al. examined the prognosis of *p-stage* T3 NSCLC with additional tumor nodules in the same lobe (T3-Add). By interrogating the SEER database and employing propensity score matching (PSM) to account for bias, their results indicated that *p-stage* T3-Add had improved survival vs. other T3 patients and similar survival to T2b patients. While the study suggests reconsideration of the T-category for T3 patients based on additional nodules in the same lobe, previous analyses by the International Association for the Study of Lung Cancer (IASLC) showed a trend toward longer OS in the T3-Add vs. T3 group but the result was not statistically significant (6) and the forthcoming proposal for the ninth edition of the TNM classification for lung cancer maintains the status quo for T3 tumors (7, 8). A further SEER analysis by Hao and Li investigated the metastatic patterns and prognosis of various subtypes of lung cancer: the liver was the most common site of metastasis for SCLC, while BMs were predominant in large cell carcinoma. Squamous cell carcinoma and adenocarcinoma showed a higher likelihood of bone metastasis. In addition, nomograms were developed to predict metastasis and survival probabilities, showing good performance in predicting distant metastasis and overall survival.

Van Dao et al. described the clinical insights into the treatment patterns in stage III non-small cell lung cancer (NSCLC) in the Vietnamese population within the KINDLE-Vietnam cohort study, claiming the firm need for guideline adoption, physician education, and multidisciplinary team in the real-world management of locally advanced NSCLC.

Historically, the 2-year incidence of BMs in stage III locally advanced (LA-)NSCLC has been estimated at 30%. However, recent clinical trials, such as PACIFIC (9) have shown a lower incidence. Although prophylactic cranial irradiation (PCI) decreased the incidence of BMs compared to observation in randomized trials, it did not translate into an overall survival benefit (10, 11). Alhusaini et al. retrospectively analyzed the incidence of BMs in their single-center cohort of 160 stage III NSCLC patients in the contemporary era of imaging. Among them, 23/160 patients (14.4%) underwent MRI surveillance after completing primary treatment while 137/160 patients (85.6%) received brain MRIs at systemic recurrence (restaging) or when neurologically symptomatic. The 2-year cumulative incidence of BMs was 17%, with a higher incidence of BMs observed in patients with adenocarcinoma and those undergoing MRI surveillance.

Velcheti et al. investigated the long-term effectiveness of single-agent pembrolizumab in patients with metastatic NSCLC, PD-L1 expression ≥50%, and good performance status (ECOG PS 0–1), confirming the consistency of RWD outcomes within real-life patients compared to those observed in controlled clinical trials (12). However, ECOG PS 2 has emerged as an independent prognostic factor with a lack of data from randomized phase III trials on the safety and efficacy of immunotherapy in this common and frail real-life setting. Yang et al. retrospectively compared the effectiveness and safety of first-line pembrolizumab vs. sintilimab, a PD-1 inhibitor approved in China, in combination with chemotherapy. The authors retrospectively analyzed data from a Chinese cohort of 164 patients with advanced squamous cell lung cancer treated between 2018 and 2022. The study demonstrated the equipoise of both regimens vis-à-vis effectiveness and toxicity in their patient population.

Liu et al. examined predictive factors and prognosis of immune checkpoint inhibitor-related pneumonitis (CIP) in advanced NSCLC. Logistic regression analysis was employed to evaluate risk factors associated with CIP. In total, 41/222 (18.5%) developed CIP and the study revealed that lower baseline hemoglobin and albumin levels were independent predictors of CIP. Furthermore, the onset of CIP was a prognosticator of overall survival in their patient cohort.

In regards to EGFR-mutant NSCLC, in the adjuvant setting, Liu et al. found out that adding chemotherapy before first-generation EGFR tyrosine kinase inhibitors (TKIs) in a Chinese cohort of stage II-IIIA patients did not improve survival compared with adjuvant EGFR-TKI alone, somewhat mirroring the recent survival data of the third-generation EGFR-TKI osimertinib, namely within the patient cohort that did not receive chemotherapy in the ADAURA trial (13) and possibly suggesting such a chemotherapy-free approach in selected low-risk patients. Moreover, in the EGFR-positive metastatic scenario, Kang et al. provided encouraging effectiveness and safety RWD in favor of the second-generation EGFR-TKI afatinib in the first-line setting of NSCLC patients with brain metastases (BMs), most importantly even in those harboring uncommon EGFR mutations. Likewise, novel prognostic models are needed in the real-world clinic to predict survival in difficult-to-treat settings such as EGFR-positive NSCLC with BMs undergoing targeted therapies (Zhu et al.).

The transformation to SCLC is a known mechanism of resistance against molecularly targeted therapies. *De novo* transformation occurs rarely, and most cases involve transformation from EGFR-mutant adenocarcinomas (14). Ding et al. investigated the effectiveness of etoposide/platinum (EP) and anlotinib plus anlotinib maintenance therapy in 10 patients with transformed SCLC from EGFR TKI-resistant lung adenocarcinomas recruited from 3 Chinese regional hospitals. This combination showed encouraging effectiveness and a low toxicity profile warranting further investigation. Chang et al. aimed to identify factors associated with outcomes after progression on first-line EGFR-TKI in advanced EGFR-mutant NSCLC patients. In total, 206/242 patients progressing on first- or second-generation TKIs and receiving second-line treatment were assessed. Second-line treatment with osimertinib was associated with longer overall survival (OS) compared to chemotherapy and other EGFR-TKIs. These findings align with the latest recommendations from the American Society of Clinical Oncology (ASCO) for patients with EGFR alterations, specifically Exon 19 deletion and L858R mutations (15).

Gow et al. retrospectively tested a large clinical cohort of NSCLC patients with no EGFR or ALK alterations using reverse transcription-polymerase chain reaction (RT-PCR) and immunohistochemistry (IHC) to detect METex14 mutations. RWD confirmed the presence of such an aggressive oncogene addiction in elderly individuals, never-smokers, with poor performance status and a higher frequency in sarcomatoid carcinoma while showing pemetrexed-based chemotherapy, strong IHC staining, BM, and lung radiotherapy being independent prognostic factors for survival in these patients. Moreover, RWE seemed to confirm the shorter median survival rates of smokers compared to non-smokers among ALK-positive patients, further suggesting the role of predictive testing irrespective of smoking status and age, as reflected by Zheng et al.

Another relevant aspect of this Research Topic addressed the real-life diagnostic setting with the seminal use of cerebrospinal fluid (CSF) as a liquid biopsy tool to complement the genomic profiling of plasma circulating tumor DNA (ctDNA) in a large cohort of NSCLC patients with brain metastases. In such a dismal setting, Shen et al. detected a higher diagnostic accuracy using whole genome sequencing on CSF supernatant compared to plasma, including all genomic alterations, especially the troublesome copy number variations.

Zhou et al. developed a prediction model using serum folate receptor-positive circulating tumor cells (FR^+^CTC) and various other blood biomarkers including tumor markers to non-invasively aid in the preoperative diagnosis of benign vs. malignant solitary pulmonary nodules (SPNs). Age, FR+CTC, thymidine kinase 1 (TK1), and neuron-specific enolase (NSE) were independently associated with malignant SPNs on multivariable analysis. They developed a predictive model incorporating these factors, achieving a sensitivity of 71.1%, specificity of 81.3%, and an area under the curve (AUC) of 0.826, demonstrating a superior performance than any single biomarker which could aid in predicting SPN malignancy.

Further, RWE is required for testing the clinical application of biosimilar drugs to reference originator products. In this vein, Zhao et al. retrospectively confirmed the effectiveness and safety of biosimilar bevacizumab in 946 Chinese patients with locally advanced or metastatic NSCLC with no new safety concerns.

Wang et al. addressed clinical and prognostic features of a rare form of adenocarcinoma, namely pulmonary enteric adenocarcinoma (PEAC) in their cohort of 26 patients recruited between 2014 and 2021. In addition, the authors interrogated the Surveillance, Epidemiology, and End Results (SEER) database which identified 20 patients. Treatment was in line with the management of lung cancer in general across all stages and prognosis was unsurprisingly determined by disease stage.

Robinson et al. addressed the issue of random missing data from a large population-based dataset of NSCLC patients in Ontario, Canada. Characteristics and outcomes of staged vs. unstaged patients were compared. In total, 51,152 patients were analyzed with 5,707 (11.2%) patients unstaged, with evidence that stage data was not missing completely at random. Unstaged patients were more likely to be older, have a higher comorbidity index, and have lower socioeconomic status. In addition, survival analysis suggested that unstaged patients had a proportion of early- and advanced-stage disease with a significant proportion likely being stage IV experiencing rapid death. The study highlights the potential bias in the evaluation of healthcare utilization and outcomes for staged patients, as unstaged patients may represent a distinct subset with different characteristics and prognoses.

## 3 Conclusions and perspectives

The landscape of lung cancer research is evolving rapidly, with a growing recognition of the crucial role that real-life data and evidence play in enhancing our understanding and management of this devastating disease. Traditional clinical trials, while invaluable, offer controlled environments that may not fully mirror the complexities of real-world patient care. By contrast, RWD directly captures insights from everyday clinical practice, providing a more comprehensive understanding of how treatments perform across diverse patient populations and clinical settings.

The editorial’s emphasis on the significance of RWE in augmenting lung cancer research is underscored by various studies presented. From investigating treatment effectiveness in metastatic NSCLC to exploring the clinical insights into treatment patterns in different populations, the evidence consistently highlights the value of RWE in filling crucial knowledge gaps and informing more effective treatment strategies.

Moreover, the editorial and associated studies shed light on several key areas of research and clinical practice, including the development of prediction models for long-term survival, the exploration of treatment approaches in specific patient subgroups, and the identification of factors associated with outcomes and prognosis.

As we move forward, it is imperative to continue integrating RWD and RWE into lung cancer research and clinical practice. This approach not only enhances our ability to tailor treatments to individual patients but also enables us to address disparities in care, optimize treatment strategies, and ultimately improve outcomes for patients battling this formidable disease. By embracing RWE, we can take significant strides toward advancing the fight against lung cancer and reducing its devastating impact on individuals and communities worldwide, often not included in randomized controlled trials.

## Author contributions

VG: Writing – original draft, Writing – review & editing. CE: Writing – review & editing.
